# A novel gap-PCR with high resolution melting analysis for the detection of α-thalassaemia Southeast Asian and Filipino β^0^-thalassaemia deletion

**DOI:** 10.1038/srep13937

**Published:** 2015-09-14

**Authors:** Siew Leng Kho, Kek Heng Chua, Elizabeth George, Jin Ai Mary Anne Tan

**Affiliations:** 1 Department of Biomedical Science, Faculty of Medicine, University of Malaya, 50603 Kuala Lumpur, Malaysia; 2 Assunta Hospital, Petaling Jaya, Selangor, Malaysia

## Abstract

Homozygosity for the α-thalassaemia Southeast Asian (α-SEA) and Filipino β^0^-thalassaemia (β-FIL) deletions can cause serious complications leading to foetal death or life-long blood transfusions. A rapid and accurate molecular detection assay is essential in populations where the deletions are common. In this study, gap-polymerase chain reaction (PCR) with high resolution melting (HRM) analysis was developed to detect both the large deletions. Melting curves at 86.9 ± 0.1 °C were generated by normal individuals without the α-SEA deletion, 84.7 ± 0.1 °C by homozygous α-SEA deletion individuals and two melting curves at 84.7 ± 0.1 °C and 86.9 ± 0.1 °C by α-SEA deletion carriers. Normal individuals without the β-FIL deletion produce amplicons with a melting temperature (Tm) at 74.6 ± 0.1 °C, homozygous β-FIL individuals produce amplicons with Tm at 73.6 ± 0.1 °C and heterozygous β-FIL individuals generate two amplicons with Tm at 73.6 ± 0.1 °C and 74.6 ± 0.1 °C. Evaluation using blinded tests on 220 DNA samples showed 100% sensitivity and specificity. The developed assays are sensitive and specific for rapid molecular and prenatal diagnosis for the α-SEA and β-FIL deletions.

Alpha- and β-thalassaemias are common autosomal recessive disorders. Alpha-thalassaemia is characterised by reduced or absence of α-globin chain synthesis and is mainly caused by deletions in the α-globin gene complex. The α-thalassaemia Southeast Asian (α-SEA) deletion (NG_000006.1:g.26264_45564del19301) removes a large sequence which includes the ψα2, ψα1, α2, α1 and θ-globin genes^1^. α-SEA deletion carriers are generally asymptomatic or show mild anaemia, however, couples who are both α-SEA deletion carriers have a 25% chance of conceiving a foetus with Hb Bart’s hydrops foetalis (−−^SEA^/−−^SEA^), a condition incompatible with life. Absence of α-globin chain production causes an imbalance production of β-globin chains which forms γ_4_ tetramers (Hb Barts). The remaining intact ζ2-gene in these foetuses maintains the production of embryonic Hb Portland (ζ_2_γ_2_) which keeps the foetus alive until around 23–38 weeks. The hydropic foetus is characterised by severe hepatosplenomegaly, hydrocephaly, hypochromic anaemia, oedema, pleural effusions and pericardial effusions^2^. In addition, serious maternal complications include placentomegaly, hypertension (50%) and maternal cardiac failure (10%). In the Malaysian Chinese and in Thailand, the α-SEA deletion is the most common defect producing α-thalassaemia, and it is also the second most common defect in the Malaysian Malays^3^.

Beta-thalassaemia is characterised by reduced or absence of β-globin chains^4^. The Filipino β^0^-thalassaemia (β-FIL) deletion (NG_000007.3:g.66258_184734del118477) removes approximately 118 kb of the β-globin gene. Patients with homozygous β-FIL deletion require life-long monthly blood transfusions due to severe anaemia and iron chelation therapies^5^ are necessary to excrete the excess iron accumulated in organs in order to increase their life expectancy^6^. The β-FIL deletion is reported as the main mutation in thalassaemia patients in the indigenous populations in Malaysia. It was the single β-globin gene defect responsible for β-thalassaemia major in 20 Dusun families in Sabah^7^. A high incidence (12.8%) of the β-FIL deletion was also reported in the Kadazandusun population^8^. In another study in the indigenous groups in Northern Sarawak, the β-FIL deletion accounted for 26/28 (93%) of the β-thalassemia alleles in transfusion-dependent thalassaemia patients^9^.

The polymerase chain reaction (PCR) is the most common method to detect the deletional thalassaemias. Gap-PCR amplifies the deleted DNA sequence using the primers flanking the deleted region^10^. Three primers are designed for each deletion to amplify the normal (undeleted) and deleted gene sequences. However, as conventional gap-PCR requires post-PCR handling and is time-consuming, it is not suitable for large-scale screening. HRM analysis is a high-throughput mutation scanning method which is based on melting temperature (Tm) profiles. The melting temperature refers to the temperature when half of the total quantity of double stranded DNA (dsDNA) have dissociated to become single stranded DNA^11^. The changes in Tm of the DNA duplexes are detected during dissociation of the dsDNA to single stranded DNA. Differences in the Tm profile enable the identification of different genotypes. HRM has various additional advantages compared with other mutation scanning methods as it can not only detect multiple known and unknown mutations, it also offers straight forward and rapid analysis. HRM is a sensitive and specific high performance platform in a close-tube system^12^,^13^. Furthermore, unknown mutations detected by HRM analysis can be directly analysed and confirmed by sequencing using amplicons obtained from the same HRM assay without any delays.

## Results

### Development of HRM analysis

Primers for HRM analysis were optimised for different annealing temperatures, primer concentrations and PCR additives. The primers amplified well at 60 °C and at primer concentrations of 5 μM. The reactions required 0.5X PCRx Enhancer to increase the specificity of amplifications. The PCRx Enhancer System consisted of optimised buffer and co-solvent which facilitated the amplification of problematic or GC-rich templates. The amplified products were electrophoresed in 1.5% (w/v) agarose gel and no primer dimers were observed.

DNA amplification was checked using the 7500 Fast Software after each run of real-time PCR. The C_T_ value for each curve was targeted to be from 20–30 to obtain optimal amplification. The amplification plot should show a steep exponential phase and a flat plateau. The melt curve analysis was performed using HRM Software version 2.0.1 (Applied Biosystems). The derivative melt curve showed the fluorescence signal at every temperature. The aligned melt curve is the normalised fluorescence curve which eliminated the differences in background fluorescence^14^. The pre- and post-melt regions were set near to the melting curve. Pre-melt refers to the temperature when initial fluorescence signals are generated and every amplicon is double-stranded. Post-melt refer to the temperature when final fluorescence signals are generated and every amplicon is single-stranded.

**Figure 1:**
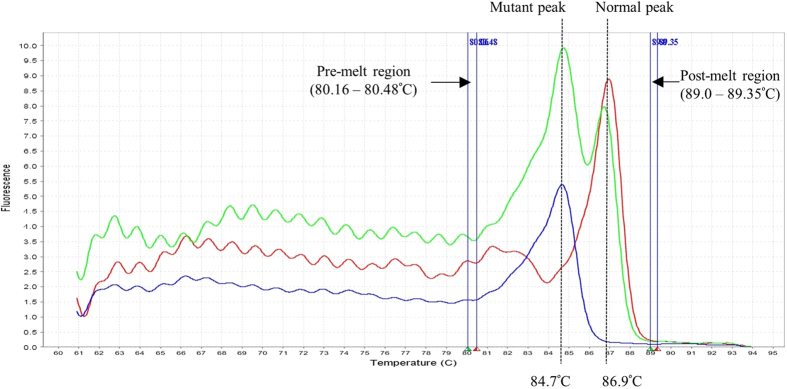
Derivative melt curves of a normal individual without the α-SEA deletion (green), α-SEA deletion carrier (blue) and DNA from homozygous α-SEA deletion (red). Pre- and post-melt regions were set manually near to the melting curves in the derivative melt curve.

Figure 1 shows the Tm and melting profiles for detection of the α-SEA deletion. DNA from normal individuals produced amplicons with a Tm at 86.9 ± 0.1 °C. DNA from homozygous α-SEA deletion individuals produced amplicons with a Tm at 84.7 ± 0.1 °C. The heterozygous DNA samples generated mutant and normal curves with Tm at 84.7 ± 0.1 °C and 86.9 ± 0.1 °C. The development of the assays using known controls showed Tm with a standard deviation of 0.1 °C due to the variations in different DNA samples.

**Figure 2:**
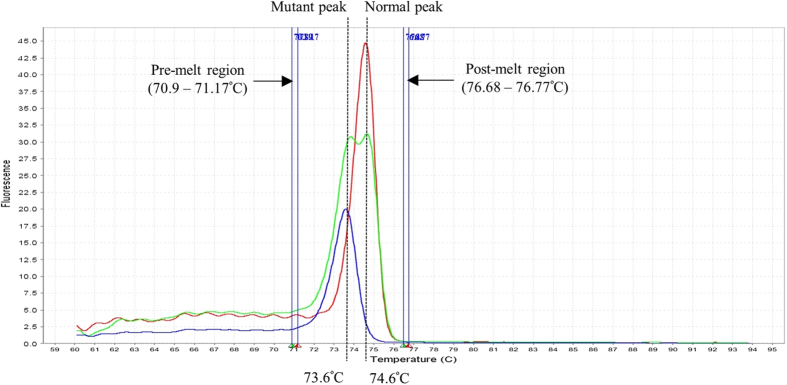
Derivative melt curves of normal individual without the β-FIL deletion (green), β-FIL deletion carrier (blue) and DNA from homozygous β-FIL deletion (red). Pre- and post-melt regions were set manually near to the melting peaks in the derivative melt curve.

Figure 2 shows the melting profiles for detection of the β-FIL deletion. DNA from normal controls produced amplicons with a Tm at 74.6 ± 0.1 °C (red curve). DNA from homozygous β-FIL individuals produced amplicons with a Tm at 73.6 ± 0.1°C (blue curve). The heterozygous β-FIL DNA samples generated 2 amplicons with Tm at 73.6 ± 0.1 °C and 74.6 ± 0.1 °C (green curve). Similarly, the known controls showed a standard deviation of 0.1 °C due to the variations in DNA samples.

### Evaluation of HRM analysis

All 220 DNA samples were accurately genotyped using HRM analysis in a blind test evaluation and the results were identical to that using the conventional gap-PCR method. The sensitivity and specificity of the developed assays were both 100% in detection of the α-SEA and β-FIL deletions. Table 1 shows the type and number of the evaluation samples and mean and standard deviation (SD) of melting temperatures (Tm).

## Discussion

Gap-PCR combined with HRM analysis was developed in this study to detect the α-SEA and β-FIL deletion. HRM analysis is a post-PCR analysis method using melting temperature (Tm) profiles to infer genotypes of individuals^15^. Using HRM analysis, DNA templates are initially amplified with forward and reverse primers, dNTP mixture, MgCl_2_ and *Taq* polymerase, followed by HRM analysis with dsDNA-binding fluorescence dyes and additional DNA melting steps. When the melting temperature is attained, the fluorescence dye is released and the fluorescence signal is decreased. Gap-PCR with HRM analysis is suitable for large-scale screening and prenatal diagnosis as it is a straight forward, cost-effective, sensitive and specific platform for molecular characterisation of mutations^16^.

Primer design for HRM assays in this study was critical for maximum sensitivity and specificity. The amplicon must be kept short (50–250 bp), as a short amplicon maximises the melting differences and increases the sensitivity of mutation detection^17^. Amplicons larger than 400 bp have been reported to have a higher error rate^18^. The genotype of an unknown DNA sample was analysed based on a similar melting profile with the positive DNA control. Thus, development of the HRM analysis using previously characterised DNA samples was performed to select the best amplifiable DNA samples to be used as positive controls^14^.

SYBR Green1 dye has been used in dissociation curve analysis in previous studies in diagnosis of α- and β-thalassaemias^19^,^20^,^21^. Although the cost of SYBR Green dye is relatively cheaper, HRM outstands SYBR Green in terms of chemistry, instrumentation and software analysis. SYTO9 dye used in this study is a brighter and saturated dsDNA binding dye. Saturated dyes are important in the melt curve analysis to avoid over estimation of melting points^22^. SYTO9 dye has superior properties in fluorescence enhancement upon binding with nucleic acids, excitation and emission spectra, DNA selectivity and DNA binding affinity^23^. HRM analysis in previous studies required a thermal cycler plus an additional HRM instrument^24^,^25^. However, in this study HRM analysis required only a single thermal cycler (7500 Fast real-time thermal cycler). In addition, the 7500 Fast real-time thermal cycler used in this study was able to collect more data points and the HRM software produced better fluorescence normalisation algorithms.

The α-SEA deletion responsible for α-thalassaemia is present in a higher frequency in Southeast Asia including Malaysia, Thailand and Singapore^3^,^26^,^27^. As homozygosity for the α-SEA deletion causes a fatal disorder, it is vital to screen for the α-SEA deletion especially in the Malaysian Chinese as this deletion is responsible for the majority of α-thalassaemia and Hb Bart’s hydrops foetalis in this race. HRM analysis detects α-SEA deletion accurately using the derivative melt curve. A previous study reported that maternal contamination in a foetal sample can be confirmed by C_T_ value discrepancy of 5 cycles in real-time PCR^28^. However, C_T_ value discrepancy was not observed in this study, indicating that maternal contamination was not present in the CV DNA samples studied. All CV samples in this study were thoroughly cleaned with sterile normal saline using a dissecting microscope before DNA extraction.

More than 200 β-globin gene defects were reported, including point mutations, addition and deletions^29^. HRM analysis was developed for detection of the β-globin gene mutations in previous studies^30^,^31^. In this study, the HRM analysis developed for detection of the β-FIL deletion showed 100% specificity and sensitivity. As the Filipino β^0^-thalassaemia deletion was reported to be present in a high frequency in the indigenous groups in East Malaysia^7^,^8^,^9^, molecular screening for this defect should be implemented in order to prevent the birth of β-thalassaemia major children. It is even more pertinent that screening for the β-FIL deletion be carried out as the majority of these individuals are not aware that they are thalassaemia carriers due to their isolation from towns and limited medical care. Marriages within individuals of the same indigenous groups are common, in addition to their practise of consanguinity. HRM analysis developed in this study was able to detect DNA samples with the β-FIL deletion accurately using the derivative melt curve. The accuracy and rapidity of this technique will allow the HRM analysis to serve as an alternative molecular approach for large-scale screening and routine diagnosis of β-FIL deletion in the Chinese and indigenous populations in Malaysia. Identified individuals can be given genetic counselling, and preventive control programmes for thalassaemia can be implemented. Thus, gap-PCR with HRM analysis allows rapid and accurate identification of two important deletional thalassaemias in Malaysia.

## Methods

### DNA samples

Couples at risk of producing a thalassaemia major child were advised to carry out prenatal diagnosis during 10–14^th^ weeks of pregnancy. After written and informed consent were obtained from the couples, blood samples and chorionic villi samples (CVS) were collected in EDTA tubes. DNA was extracted using conventional phenol-chloroform method^8^. Seventy-five DNA samples were previously characterised using gap-PCR and used in the development of HRM analysis (Table 2). Ethics approval was obtained from the Medical Ethics Committee of University Malaya Medical Centre (MEC:344.7) in accordance with the Declaration of Helsinki.

### Gap-PCR

Gap-PCR was performed to detect the α-SEA and β-FIL deletions using a Veriti thermal cycler (Applied Biosystems, USA). The total reaction volume for gap-PCR was 25 μl which contained 10X PCR buffer, 2.5 U *Taq* DNA polymerase (Fermentas, Germany), 800 μM of dNTP, 20 pmol for each primer, 2.5 mM magnesium chloride (MgCl_2_)^7^,^9^. The cycling conditions for the α-SEA deletion involved 95 °C for 5 minutes, followed by 30 cycles of denaturation at 93 °C for 1 minute, annealing at 60 °C for 1 minute and extension at 72 °C for 1.5 minutes, and finally an extension step at 72 °C for 6 minutes. The cycling conditions for β-FIL deletion involved 35 cycles of denaturation at 95 °C for 1 minute, annealing at 60 °C for 1 minute and an extension at 72 °C for 1 minute. The PCR products were resolved in a 1.5% (w/v) agarose gel.

### Primer design for HRM analysis

**Figure 3:**
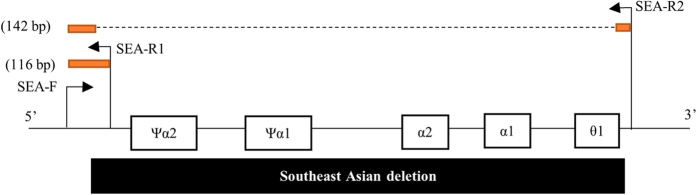
The position of forward (SEA-F) and reverse primers (SEA-R1 and SEA-R2) in the α-globin gene complex. The black box indicates the 19 kb α-thalassaemia Southeast Asian deletion (−−^α-SEA^). The orange boxes indicate the PCR products.

The genes and regions of interest were identified using the Basic Local Alignment Tool (BLAST). Primers were designed to amplify the α-SEA deletion using Primer Express Software (Applied Biosystems). A common forward primer (SEA-F: 5′-CGCAGGAACTCGGTCGTC-3′) and two reverse primers (SEA-R1: 5′-ACTGCTCCGCTCCACCCG-3′ and SEA-R2: 5′-GTAGTCATGGCTTACTGCAGC-3′) were designed (Fig. 3). The primer sequences were tested using *in silico* PCR. Differences between the amplified products (26 bp) generated two Tm in order to identify the genotype of an individual. DNA from a normal individual without the α-SEA deletion generated an amplicon of 116 bp, DNA from a Hb Bart’s hydropic foetus produced a PCR product of 142 bp and α-SEA deletion carriers produced PCR products of 116 bp and 142 bp.

**Figure 4:**
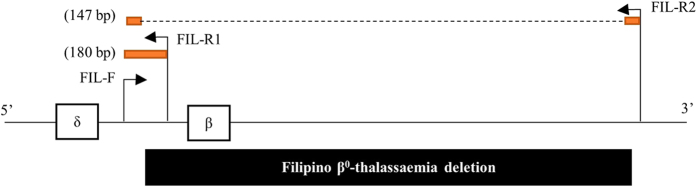
The position of forward (FIL-F) and reverse primers (FIL-R1 and FIL-R2) in the β-globin gene. The black box indicates the Filipino β^0^-thalassaemia deletion. The orange boxes indicate the PCR products.

Primers were designed to amplify the β-FIL deletion using Primer Express Software. A common forward primer (FIL-F: 5′-GGAGATACTTGTGTGGTATTCGAAAG-3′) and two reverse primers (FIL-R1: 5′-CCAACATCTTGCCTAGACTCACTG-3′ and FIL-R2: 5′- TGCAGAAAAAGACAGTTGGACTTAA-3′) were designed to amplify products of 180 bp and 147 bp (Fig. 4). The primer sequences were tested using *in silico* PCR. Differences between the amplified products (33 bp) enabled the identification of genotype of an individual. DNA from a normal individual without the β-FIL deletion generated an amplified product of 180 bp, DNA from homozygous β-FIL individuals developed a PCR product of 147 bp and β-FIL deletion carriers produced PCR products of 147 bp and 180 bp.

### Optimisation of HRM analysis

Specific and robust PCR was developed using a gradient thermal cycler. Optimisation was carried out with different annealing temperatures from 55 °C to 65 °C. Optimisation of HRM analysis was carried out using different primer concentrations ranging from 3 μM to 8 μM. Magnesium chloride at different concentrations ranging from 1.5 mM to 3.0 mM were added for PCR optimisation. In addition, dimethyl sulfoxide (DMSO) (Sigma) and PCRx Enhancer (Invitrogen) were also used in the optimisation of HRM analysis.

### Real-time PCR with HRM analysis

Real-time PCR with HRM analysis was performed in a total volume of 10 μL using a 7500 Fast Real-time PCR thermal cycler (Applied Biosystems). The reactions contained 5 μL of MeltDoctor^TM^ HRM Master Mix (Applied Biosystems), 0.6 μL of each forward and reverse primer, PCR additives, 1 μL of DNA and topped up to 10 μL with double-distilled water. The thermal cycling conditions involved enzyme activation at 95 °C for 10 minutes, followed by 40 cycles of denaturation at 95 °C for 15 seconds and 1 minute of annealing/extending at 60 °C. For the melt curve analysis, the thermal cycling conditions started with denaturation at 95 °C for 10 seconds, annealing at 60 °C for 1 minute, HRM at 95 °C for 15 seconds and finally annealing at 60 °C for 15 seconds. DNA amplification was checked using Applied Biosystems 7500 Software version 2.0.6. The melt curve analysis was performed using the Applied Biosystems HRM Software version 2.0.1.

### Evaluation

The developed HRM analysis was further evaluated to determine its sensitivity and specificity. DNA samples (n =220) used in this study consisted of 38 DNA samples from normal individuals and 182 DNA samples from thalassaemia carriers and major patients.

## Additional Information

**How to cite this article**: Kho, S. L. *et al.* A novel gap-PCR with high resolution melting analysis for the detection of α-thalassaemia Southeast Asian and Filipino β^0^-thalassaemia deletion. *Sci. Rep.* **5**, 13937; doi: 10.1038/srep13937 (2015).

## Figures and Tables

**Table 1:** Type and number of evaluation samples and the mean and standard deviation (SD) of the melting temperature (Tm).

Deletion type	Sample type	Number ofsamples	Tm mean ± SD (°C)
α-SEA	α-SEA deletion negative	215	86.9 ± 0.1
	α-SEA deletion carrier	5	84.7 ± 0.1 and 86.9 ± 0.1
	Homozygous α-SEA deletion	0	−
β-FIL	β-FIL deletion negative	218	74.6 ± 0.1
	β-FIL deletion carrier	2	73.6 ± 0.1 and 74.6 ± 0.1
	Homozygous β-FIL deletion	0	−

**Table 2:** List of DNA samples used in the development of high resolution melting analysis for detection of the α-thalassaemia Southeast Asian (−−^α-SEA^) and Filipino β^0^-thalassaemia (β-FIL) deletions.

Thalassaemia status	Number
Normal	25
α-SEA deletion carrier	23
Homozygous α-SEA deletion	4
β-FIL deletion carrier	21
Homozygous β-FIL deletion	2
TOTAL	75
